# Hilling as a cultural control strategy for soybean gall midge (Diptera: Cecidomyiidae)

**DOI:** 10.1093/jee/toad195

**Published:** 2023-10-25

**Authors:** Anthony Justin McMechan, Joana Schroeder de Souza, Natasha Umezu, Pragya Gupta, Gabriela Inveninato Carmona

**Affiliations:** Department of Entomology, University of Nebraska-Lincoln, NE 68583, USA; Department of Entomology, University of Nebraska-Lincoln, NE 68583, USA; Department of Entomology, University of Nebraska-Lincoln, NE 68583, USA; Department of Entomology, University of Nebraska-Lincoln, NE 68583, USA; Department of Entomology, University of Nebraska-Lincoln, NE 68583, USA

**Keywords:** soybean gall midge, hilling, soybean, IPM

## Abstract

Soybean gall midge, *Resseliella maxima* Gagné, was recently identified as a new species causing significant injury to soybean and is currently found in 164 counties across 7 midwestern states (NE, IA, SD, MN, MO, ND, and KS). Infestation of soybean begins in late spring, when adults emerge from last year’s soybean field. Infestation of a new soybean crop depends on the presence of fissures which start to form at the base of the soybean plant around the V2 stage. Field observations indicate that these fissures are only present below the cotyledonary nodes or in the area within 3–5 cm above the soil surface. To determine the importance of these fissures for *R. maxima* infestation and plant injury, hilling or the movement of the soil to cover the base of soybean plants at the V2–V3 stage was compared with the standard practice (no-hilling). Field studies were conducted at 3 sites in east-central Nebraska during the 2021 growing season. The results showed a significant reduction in the frequency of infested plants, larval number per plant, and plant injury for hilled compared to no-hill treatment. This reduction in the presence of larvae and plant injury corresponded with a significantly greater yield for hilled compared to the no-hill treatment. These results highlight the importance of fissures on soybean for *R. maxima* adult infestation as well as the potential for hilling to be used as a management strategy for *R. maxima*.

## Introduction

Soybean gall midge (*Resseliella maxima* Gagné) was found to be associated with dead and dying soybean plants in the summer of 2018 (Gagné et al. 2019). Later in 2019, it was identified as a new species posing a potential threat to the crop. *Resseliella maxima* overwintering adults emerge from last year’s soybean field in late spring and move to the closest soybean field, resulting in plant injury starting from the field border. McMechan et al. (2021a) reported a heavily infested field at intervals from the field edges. The first 30 m recorded yield losses greater than 92%, followed by 31% and 20% at 60 and 120 m away from the edge of the field, respectively. The extensive plant injury and significant losses from *R. maxima* have resulted in several field studies to identify potential management strategies for the pest.

Several attempts have been made to identify an effective management strategy for *R. maxima*. To date, only chemical control strategies have been published for *R. maxima.* In 2020, a study applying Thimet 20G (a.i.: phorate) showed a significant reduction in larval number, plant injury, and increased yield compared to untreated plots (McMechan 2021). Unfortunately, the use of Thimet 20G is limited due to the need for specialized equipment. Foliar insecticides have not been effective against *R. maxima*, likely because adult emergence from overwintering sites is over a long duration (Hodgson and Helton 2021, Montenegro 2022). As a result, there is a growing need for alternative, non-chemical control strategies for *R. maxima*.

Hilling is the use of tools such as disks, sweep shovels, or similar equipment that move the soil between plant rows and deposit it beside or on top of the row after planting (Carling and Walworth 1990). In potatoes, hilling can be used to control weeds, improve drainage, or raise soil temperatures (Carling and Walworth 1990). Saffigna et al. (1976) reported that hilling could result in uneven water availability and fertilizer loss due to increased leaching in potato fields. The effect of tillage on soybean yields has been studied extensively in the past, with differences in early-season growth (Yusuf et al. 1999); however, no significant differences in yield were observed with adequate early-season moisture (Elmore 1987). Hilling, as applied in this study, can be confused with ridge tillage where seeds are sown on an elevated seedbed prepared above the normal land surface of the field. In the case of ridge tillage, the base of the stem is not covered with soil (Hatfield et al. 1998), while in hilling, the base of the plants is covered with soil.

**Fig. 1.**
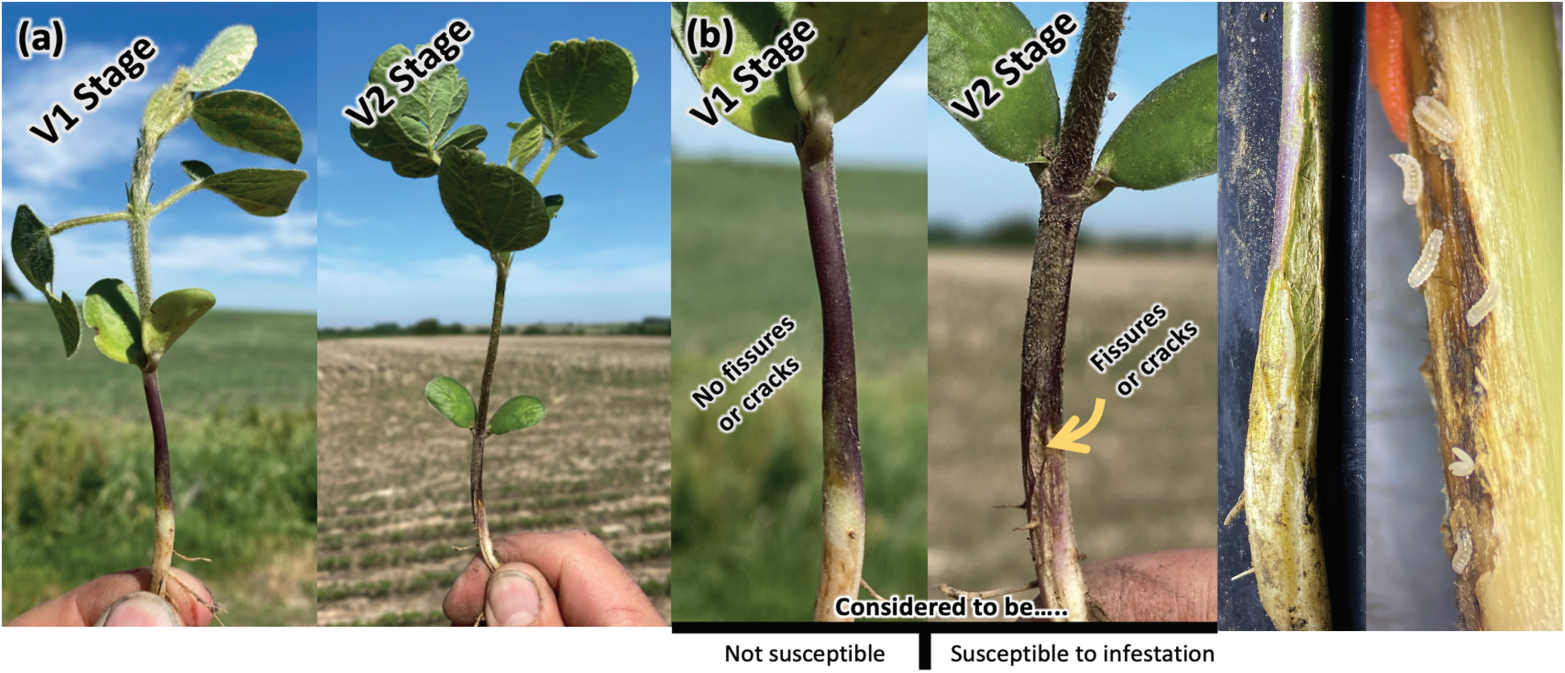
a) Soybean at V1 and V2 stages and natural b) soybean fissures or cracks during V1 (not susceptible to *R. maxima* and V2 [susceptible to soybean gall midge]) stages.

The use of hilling is not common for soybean production in the Midwest. However, limiting the ability of *R. maxima* to lay eggs and infest the soybean plants through a physical soil barrier could reduce yield losses. As a recently identified species there is little knowledge about its behavior, but past observations across multiple states, *R. maxima* starts infesting the soybean plants at the V2 or V3 growth stages when natural fissures below the cotyledonary node begin to appear in soybean (Fig. 1; McMechan et al. 2021b). Some greenhouse observations with a time-lapse camera show adults laying eggs in the soybean plant fissures (McMechan et al. 2021b). After the eggs hatch, larvae feed on tissue in the base of the stem starting from the phloem and moving to the xylem (Gagné et al. 2019). Larvae are white (first and second instar) and orange (third instar) in color. To evaluate the hypothesis that fissures are crucial for *R. maxima* infestation, fissures were covered with soil using the hilling technique in a field experiment. A significant reduction in *R. maxima* larval number, soybean injury, and greater grain yield was expected in plots where hilling was used. Understanding the role of fissures for *R. maxima* infestation is crucial for developing future management practices. In addition, chemical control strategies have shown limited efficacy against *R. maxima*. Identifying other possible effective measures, such as hilling, will provide growers with alternative management strategies.

## Materials and Methods

### Study Description

**Fig. 2.**
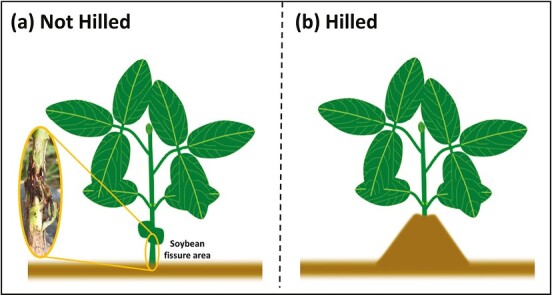
Study treatment differences illustration. a) Not hilled does not cover the soybean natural fissure area, where *R. maxima* is expected to lay eggs, while b) hilled covers the soybean natural fissure area with soil.

Field studies were conducted at 3 Nebraska sites (Lancaster, Cass, and Otoe County) in 2021 to evaluate the efficacy of hilling as a cultural control on *R. maxima* infestation, plant injury, and yield. The study was conducted as a randomized complete block design with repetitions varying across sites (Table 1). The same 2.8 maturity group soybean variety was bulk planted on 7th, 8th, and 13th of May for Otoe, Lancaster, and Cass County sites, respectively. At each site, soybean was seeded at a depth of 3.8 cm at 365,500 seeds/hectare in 0.76 m rows, with each plot consisting of 2 rows. The treatments included hilling and no-hilling as a check (Fig. 2). Hilling applications were made at each site around the V1–V2 stage when the plants develop fissures and are more likely to be susceptible to infestation from *R. maxima* (McMechan et al. 2021a; Fig. 1). At the Lancaster Co. site, hilling treatments were applied by hand as the plants reached the V2 stage. The soil was first broken up between the rows using a garden hoe and then moved to the row to cover the stems up to the unifoliate nodes. In contrast, hilling treatments at Cass and Otoe Co. sites were applied using a Hillside Cultivator Co. LLC Model Channel Slide with front-mounted disc gangs, middle-mounted s-tines, and rear-mounted rolling spiders. Disc gangs and rolling spiders were angled to pull soil into the soybean row to cover the soybean stem up to the unifoliate node. Rows were inspected after hilling to ensure good coverage on the stems. The number of replications, planting date, plot length, site information, sampling dates, and method of hilling varied between sites (Table 1). Adult *R. maxima* emergence was monitored in overwintering sites beginning on 1st May.

**Table 1.** Characteristics of each field site for planting date, number of replications, field characteristics prior to treatment, treatment method, sampling date, and plot length

Field site	Planting date	Reps	Field preparation	Hilling method	Hilling date	Hilling at soybean stage	Larval sample date	Plot length (m)
Lancaster	8th May	6	Heavy disc	By hand	6th June	V2	2nd July	3.3
Cass	7th May	4	No-till	Tillage unit	7th June	V2	2nd July	6.1
Otoe	13th May	8	Spring disc	Tillage unit	7th June	V1/V2	22nd June	7.6

### Samples and Evaluations

In late June and early July, a total of 10 plants were collected from each row and evaluated for the presence or absence of *R. maxima* larvae in field. The outer tissue below the cotyledonary nodes was carefully removed and examined for larvae. Of the 10 plants, 3 infested stems were randomly selected from each row for larval counts. Each stem was cut just below the soil surface and above the unifoliate node. In the case of hilling, the soil was removed from the base of the plants, and plants were cut at the same height as the no-hilled treatments. The 3 infested stems collected from each row were placed in a separate 50-ml conical tube that was later dissected to count all white and orange larvae. Plant injury scores, as described by Helton et al. (2022), were taken on each plot on the same day as plant collection for infestation evaluation and continued in 10-day intervals until late August. The area under the severity progress curve (AUSPC) was calculated as described by Helton et al. (2022). Yields were obtained with Zurn 150 combine with a straight-cut header. All yields were corrected to 13% moisture prior to any statistical analysis.

### Statistical Analysis

An analysis of variance using Generalized Linear Mixed Models (PROC GLIMMIX) was used to analyze the average larval number per plant, frequency of infested plants, AUSPC, and grain yield for each site. The average larval number was analyzed following a negative binomial distribution with a log function in SAS (SAS Institute 9.4). The frequency of infested plants was analyzed with beta distribution as values were limited between 0 and 1. Studentized residual panels showed a Gaussian distribution for both AUSPC and yield. Tukey adjustment was used on pairwise-comparison tests to control for Type I error rates. Tukey LSD is reported at α = 0.10 significance level. Due to the different application methods, plot size, and sampling time, each site was analyzed separately.

## Results

*Resseliella maxima* adults emerged at all sites in early June, and significant pressure in terms of larval number per plant, and plant injury was observed at all 3 sites. Soybean plants were all at the susceptible V2 stage when the first adult emergence occurred from overwintering sites.

### Proportion of Plants Infested and Larval Counts

Significant infestation occurred at all three sites with no-hill treatments ranging from 46% to 90% of plants infested with 7.3–21.2 total larvae per plant. A significant reduction in the frequency of infested plants with hilling compared to no-hill treatments occurred at Lancaster (*F* = 9.17; *df* = 1,5; *P* = 0.0291), Cass (*F* = 200.35; *df* = 1,3; *P* = 0.0008), and Otoe (*F* = 14.92; *df* = 1,7; *P* = 0.0062) sites (Table 2). White larval number per plant was reduced with the hilling treatment at Otoe (*F* = 15.3; *df* = 1,7; *P* = 0.0058) and Lancaster (*F* = 5.69; *df* = 1,5; *P* = 0.0628) (Table 2). A reduction in larval number was observed for orange larvae at Lancaster (*F* = 27.78; *df* = 1,5; *P* = 0.0033) and Otoe (*F* = 23.27; *df* = 1,7; *P* = 0.0019) (Table 2). In addition, the total larval number per plant was reduced for the hilling treatment at Lancaster (*F* = 20.47; *df* = 1,5; *P* = 0.0063) and Otoe (*F* = 28.22; *df* = 1,7; *P* = 0.0011) (Table 2). White, orange, and total larval number per plant could not be analyzed at the Cass site due to an absence of larvae on hilled treatments (Table 2).

**Table 2.** Frequency of infested plants and the average number of white, orange, and total larvae per plant per site from no-hilled and hilled treatments. Letters indicate significant differences at *P* < 0.10

Site	Frequency of infested plants^a^		White larvae		Orange larvae		Total larvae	
	No-hill	Hilled	No-hill	Hilled	No-Hill	Hilled	No-hill	Hilled
Lancaster	46.6% A	15.0% A	2.4 A	0.1 B	18.8 A	2.1 B	21.2 A	2.3 B
Cass^b^	87.5% A	0.0% B	1.1	0.0	6.2	0.0	7.3	0.0
Otoe	50.0% A	20.0% B	5.4 A	0.4 B	9.3 A	1.1 B	14.7 A	1.5 B

^a^Statistical comparisons were made within each site to compare no-hill and hilled treatments.

^b^No statistical analysis was conducted on orange, white, or total larvae at the Cass County site due to an absence of larvae in hilled treatments.

### Plant Injury Scores and Grain Yield

**Fig. 3.**
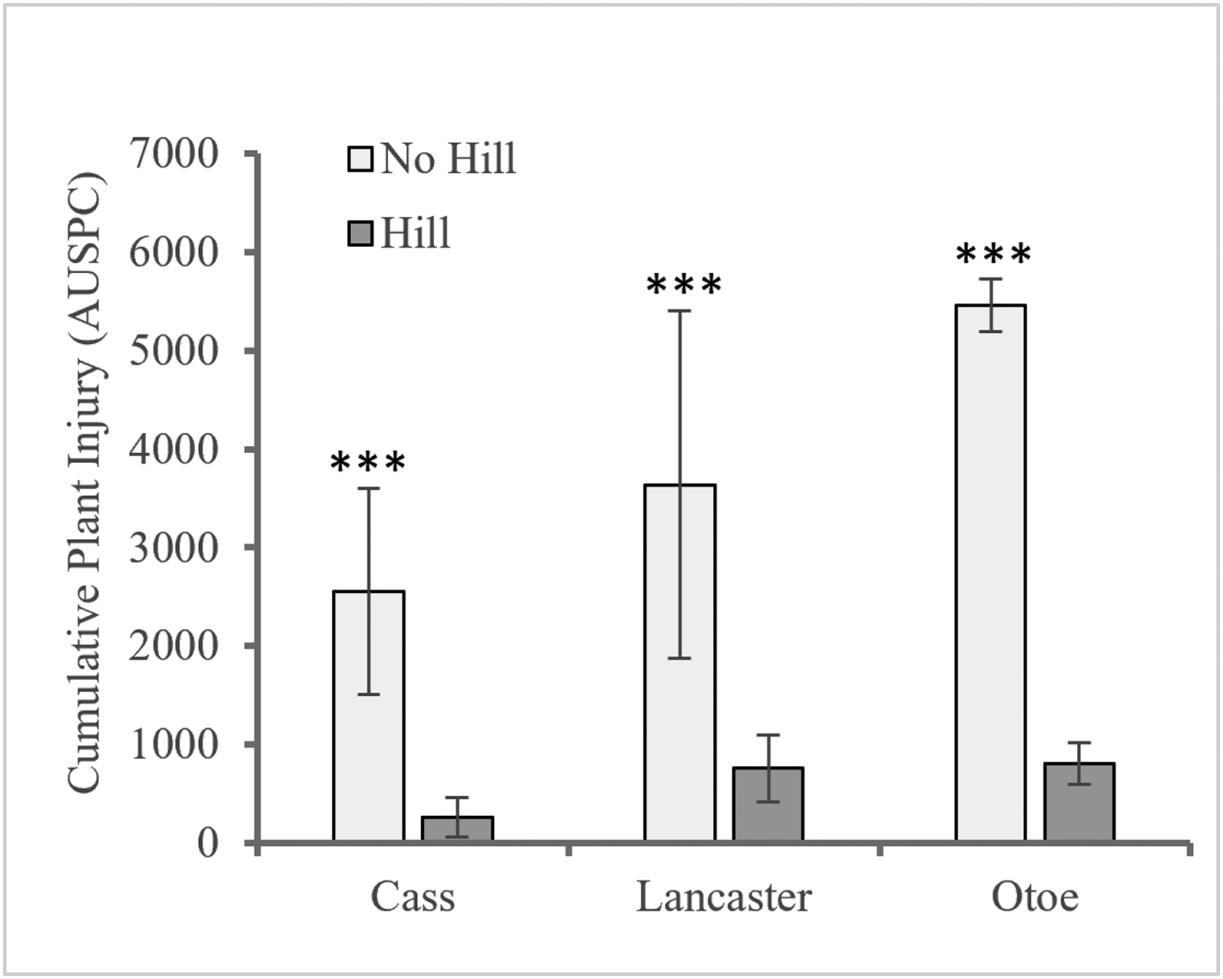
Cumulative area under the severity progress curve (AUSPC) values analysis within each site for no-hill and hilled treatments at Cass, Lancaster, and Otoe sites. *** ≤0.0001, ** ≤0.01, * ≤0.10 significant levels.

**Fig. 4.**
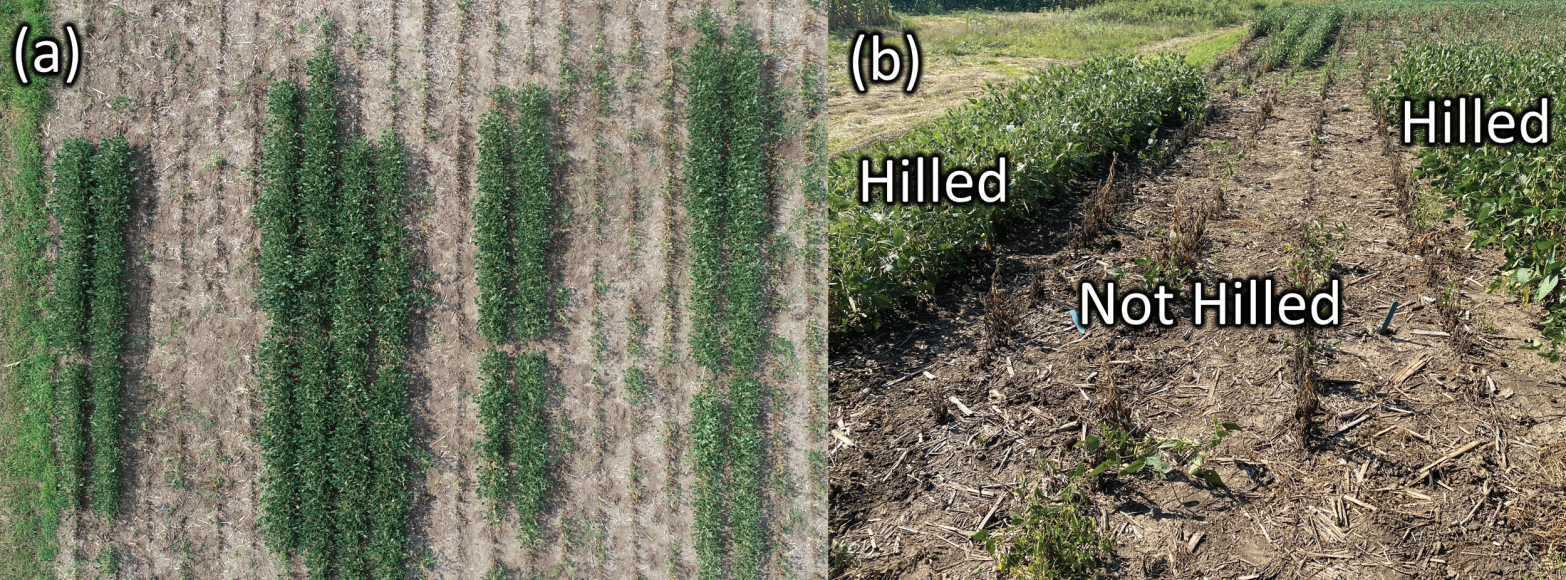
Hilled and not hilled treatment differences. a) Aerial photo taken on June 29th and b) picture taken on August 22nd from Otoe Co. Picture taken by Elliot Knoell and Justin McMechan.

Cumulative season-long AUSPC values showed that plant injury was greatest at Otoe (3,133.4), followed by Lancaster (2,198.9), and Cass (1,410.0) (Fig. 2). Hilling (760.0, 265.7, and 808.6) resulted in a significantly lower AUSPC than no-hill (3,637.9, 2,554.7, and 5,458.3) at Lancaster (*F* = 12.66; *df* = 1,5; *P* = 0.0163), Cass (*F* = 21.05; *df* = 1,3; *P* = 0.0194), and Otoe (*F* = 1,696.90; *df* = 1,7; *P* < 0.0001), respectively. Grain yield differences between treatments corresponded with plant injury scores (Fig. 3). Hilling (2,655.5, 3,886.9, and 3,111.4 kg/hectare) yielded significantly more than no-hill (341.3, 1,736.3, and 147.4 kg/hectare) for Lancaster (*F* = 580.82; *df* = 1,3; *P* = 0.0002), Cass (*F* = 99.80; *df* = 1,3; *P* = 0.0021), and Otoe (*F* = 914.13; *df* = 1,7; *P* < 0.0001), respectively. Visual differences between treatments were obvious, as shown in Fig. 4.

## Discussion

**Fig. 5.**
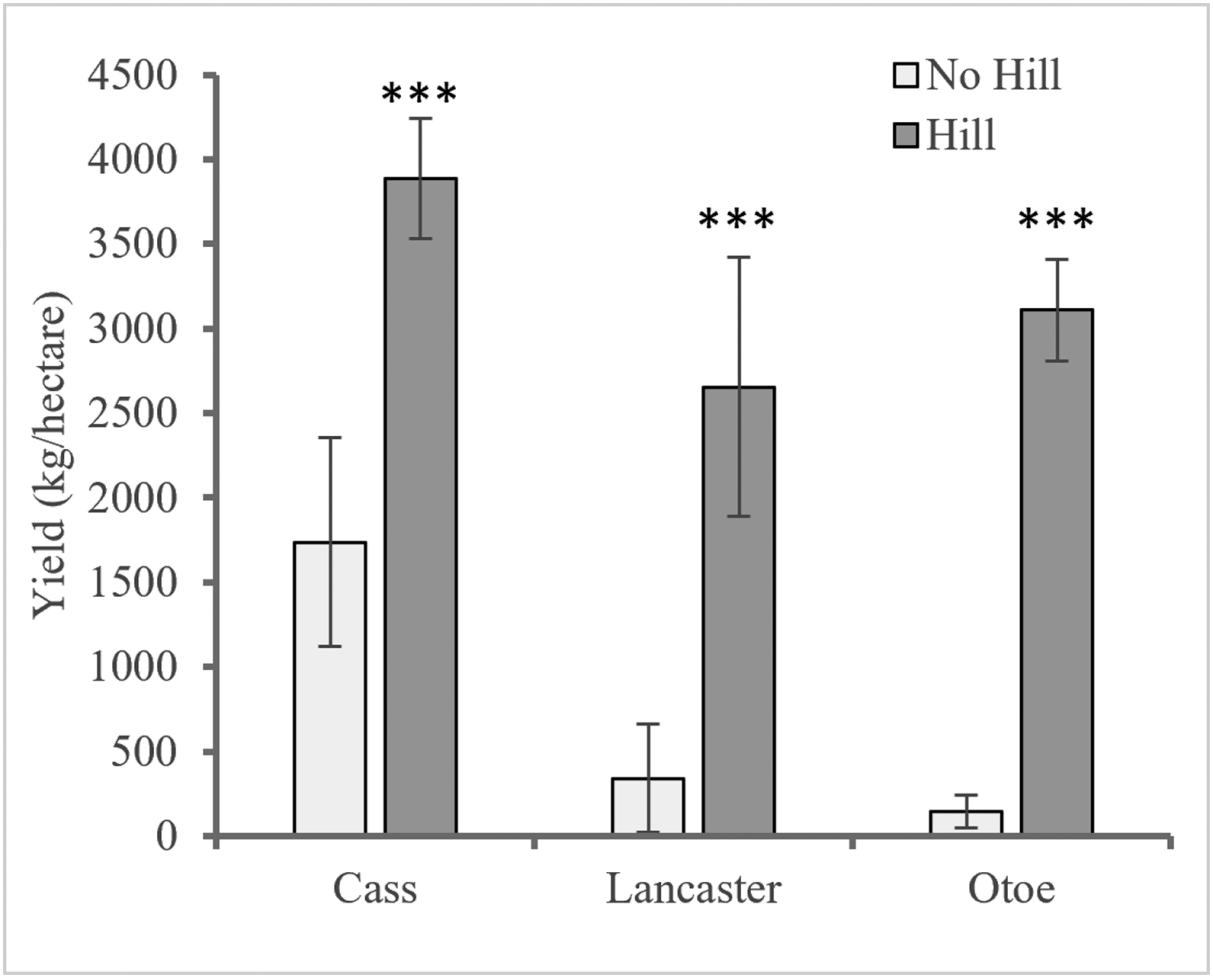
Grain yield (kg/hectare) comparison within each site for no-hill and hilled treatments at Cass, Lancaster, and Otoe sites. *** ≤0.0001, ** ≤0.01, * ≤0.10 significant levels.

As a recently identified pest causing significant yield losses in soybeans in the midwestern United States, there are limited management strategies to manage *R. maxima*. This research is the first to evaluate hilling as a possible *R. maxima* management strategy and provide crucial information regarding the role of the soybean plant fissures in the success of *R. maxima* infestation. Results from this multi-site study showed significant *R. maxima* pressure occurred at all three sites through the frequency of infested plants (Table 2), larval number per plant (Table 2), plant injury (Fig. 3), and yield (Fig. 5). Except for the proportion of infested plants at the Lancaster site, all other response variables showed a significant reduction in the proportion of infested plants, larval counts, and plant injury with a corresponding increase in yield with hilling compared to the no-hill treatment. These results indicate the potential for a hilling strategy to reduce *R. maxima* injury, infestation, and yield loss.

Besides demonstrating the effectiveness of hilling as potential management to control *R. maxima*, this study indicates the importance of soybean fissures at the base of the stem for *R. maxima* infestation and injury. Observations made by McMechan et al. (2021) indicated that *R. maxima* start to infest soybean plants at V2–V3 growth stages when natural fissures begin to develop (Fig. 1). Our results support this hypothesis; when the base of the plant was covered with soil (hilling), a physical barrier was created around the fissures that limited *R. maxima* ability to infest the plants, resulting in a lower larval number, infestation, and relatively higher soybean yield.

Hilling has been used as a management tool in potatoes (Carling and Walworth 1990), but this is the first study focusing on *R. maxima* management in soybean using hilling as a potential management strategy. However, hilling is not commonly used in soybean systems. The practice of hilling can cause soil disturbance, resulting in root damage and increased susceptibility to soil erosion (Nyawade et al. 2018). Despite the lack of use of this practice in soybean, the results obtained in this research contribute to a better understanding of *R. maxima* infestation and can also provide farmers with an alternative management strategy that can be implemented as part of an integrated pest management program.

Since *R. maxima* was recently identified as a new species and hilling is a non-common practice in soybean, there is limited literature information available that could support our findings of the methodology used in this study. As a result, there are some factors that were not covered in this experiment but should be explored in future research, such as evaluating the timing of hilling relative to plant stage or adult emergence. In addition, *R. maxima* is a pest that primarily infests field borders. Future research should evaluate if hilling only the field borders would be an appropriate management strategy or if it would result in *R. maxima* infestation further into the field interior.
